# The World of Immunization: Achievements, Challenges, and Strategic Vision for the Next Decade

**DOI:** 10.1093/infdis/jiab284

**Published:** 2021-09-30

**Authors:** Ann Lindstrand, Thomas Cherian, Diana Chang-Blanc, Daniel Feikin, Katherine L O’Brien

**Affiliations:** 1 Immunization Vaccines and Biologicals Department, World Health Organization, Geneva, Switzerland; 2 MM Global Health Consulting, Geneva,Switzerland

**Keywords:** challenges, decade, EPI, history, IA2030, immunization, impact, overview, vaccines, vision

## Abstract

Immunization is among the most cost-effective public health interventions available and is estimated to have averted at least 37 million deaths between 2000 and 2019. Since the establishment of the Expanded Programme on Immunization in 1974, global vaccination coverage increased and the coverage gap between rich and poor countries decreased. Creation of Gavi, the Vaccine Alliance, in 2000 allowed the poorest countries in the world to benefit from new, life-saving vaccines and expand the breadth of protection against an increasing number of vaccine-preventable diseases. Despite this progress, inequities in access to and uptake of vaccines persist. Opportunities to realize the full potential of vaccines are within reach but require focused, tailored and committed action by Governments and immunization stakeholders. The Immunization Agenda 2030 provides a framework for action during the next decade to attain a world where everyone, everywhere, at every age fully benefits from vaccines for good health and well-being.

## INTRODUCTION

Immunization is one of the most cost-effective public health preventive measures in countries of all income levels [1–4]. Six vaccine-preventable diseases constituted the initial focus of the Expanded Programme on Immunization (EPI) over 4 decades ago; at least 6 new vaccines have increased the breadth of protection provided by immunization, contributing to reductions in morbidity and mortality across the life course. In the midst of the challenges facing immunization programs during the COVID-19 pandemic, this article gives a historic overview of immunization, its achievements, current challenges, and introduces the new strategy and vision for the next decade, the Immunization Agenda 2030 (IA2030) [5].

### Establishment of the Expanded Programme on Immunization

The global scaleup of immunization programs began with the establishment of EPI in 1974 by the World Health Assembly (WHA) [6]. The adoption of the resolution (WHA27.57) establishing EPI was made possible due to the successes achieved under the smallpox eradication program [6].

**Figure 1.**
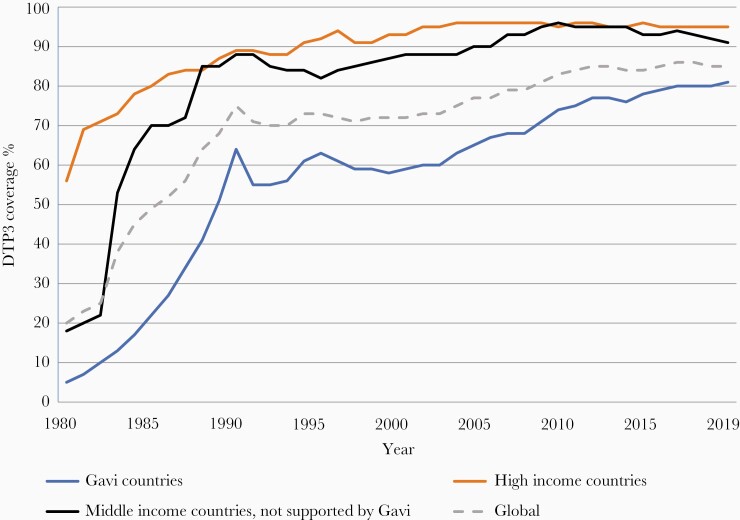
Three doses of diphtheria-tetanus-pertussis vaccine (DTP3) coverage by country income level, 1980 to 2019. World Health Organization United Nations Children’s Fund estimates of national immunization coverage.

Initial progress was slow and by 1980, coverage with 3 doses of diphtheria-tetanus-pertussis (DTP)-containing vaccines (DTP3) globally was around 20% with a wide disparity in coverage between high- and low-income countries; DTP3 coverage in the poorest countries was 5% (Figure 1). In response, in 1984, the Universal Childhood Immunization initiative led by the United Nations Children’s Fund (UNICEF) in collaboration with the World Health Organization (WHO) was established, with the ambitious aim of increasing DTP3 coverage to 80% globally by 1990. Not only did global DTP3 coverage nearly quadruple over the course of a decade, reaching 75% by 1990, but in the poorest countries rose from 5% in 1980 to 62% in 1990 (Figure 1), substantially diminishing inequity with wealthy countries.

The achievements of the EPI, along with the eradication of smallpox in 1980, laid the ground for launching aspirational vaccine-preventable disease goals over the next 20 years, including the eradication of poliomyelitis, elimination of maternal and neonatal tetanus, and elimination of measles and rubella in all WHO regions. Surveillance to document progress towards eradication and elimination goals led to the initiation and strengthening of communicable disease surveillance, especially in low- and middle-income countries (LMICs), which has served to detect and respond to epidemics of both vaccine-preventable and other communicable diseases for the past 40 years.

**Figure 2.**
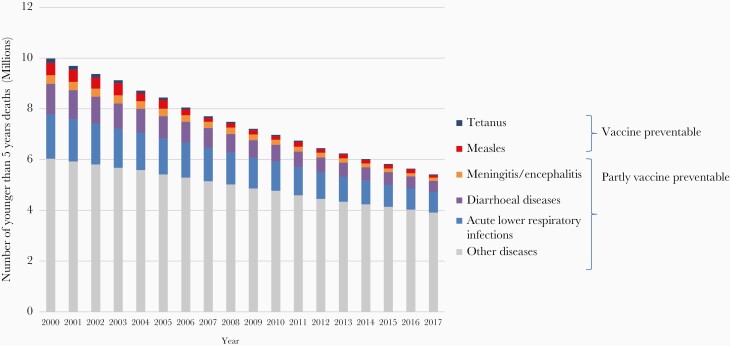
Global numbers of child deaths preventable or partially preventable through vaccination, 1990–2017. Source: WHO, Global Health Observatory data, November 2018.

Immunization has contributed to Millennium Development Goal 4 to reduce childhood mortality. All-cause mortality in children younger than 5 years decreased by 47% between the year 2000 (9.7 million deaths) and 2019 (5.2 million deaths) [7]. Four of the top 10 causes contributing to this decrease are fully or partially vaccine-preventable (ie, pneumonia, diarrhea, measles, and meningitis; Figure 2) [8]. It is estimated that vaccination has averted at least 37 million deaths (95% credible interval 30–48) between 2000 and 2019, representing a 45% decline in deaths due to 10 vaccine-preventable diseases (*Haemophilus influenzae* type b [Hib], Japanese encephalitis, *Neisseria meningitidis* serogroup A, measles, *Streptococcus pneumoniae,* rotavirus, rubella, and yellow fever) in 98 LMICs relative to no vaccination [9]. Additional deaths averted over a lifetime in the birth cohorts vaccinated from 2000 to 2030, including for hepatitis B and human papilloma virus (HPV), increase that to 120 million (93 to 150). The total annual global deaths averted by vaccination go far beyond these estimates as only 98 countries were modeled, and deaths averted from polio, diphtheria, tetanus, pertussis, and from adult vaccinations were not modelled [10]. In a related modeling study it was estimated that from 2011 to 2030, immunization would avert $1510.4 billion (2018 USD; 95% CL $674.3–$2643.2) in costs of illness in 94 LMIC compared with no vaccination, and generate $3436.7 billion (95% CL $1615.8–$5657.2) in benefits [11], representing a return of $26.1 for every dollar invested using the cost-of-illness approach [4].

### Gavi, the Vaccine Alliance

In the 1990s, immunization coverage stagnated and the slow introduction into lower-income countries of new life-saving vaccines, which were developed and implemented in many high-income countries (HICs), created serious inequities in protection from infectious disease threats to infant and child survival. In response, the Children’s Vaccine Initiative (CVI), an alliance of United Nations agencies, private foundations, and industry, was launched in 1990 with a vision that all children would be immunized and set 3 goals of vaccine supply, new vaccine development, and improved vaccine delivery [12]. However, the urgency to accelerate introduction of already available life-saving vaccines in low-income countries led to the dissolution of CVI in 1999 and the establishment of Gavi, the Vaccine Alliance (Gavi) in 2000. Founded by the Bill and Melinda Gates Foundation and key partners (WHO, UNICEF, and the World Bank), Gavi was established as a private-public partnership to catalyze the introduction of new and underused vaccines in the world’s poorest countries. Over the past 2 decades, financial and technical support from Gavi has led to the uptake of hepatitis B, Hib, yellow fever, pneumococcus, rotavirus, inactivated polio, and HPV vaccines in the world’s poorest countries, reducing the time lag between their introduction in high-income and in low-income settings.

Until 2020, Gavi approached country eligibility for support through the specification of economic status; those with per capita gross national income (GNI) below an established threshold over the preceding 3 years (according to the World Bank data published each July) can seek support for the introduction of new or underused vaccines, as well as for health systems strengthening funds for vaccine delivery infrastructure. Eligibility was set at an amount of US$1500 in 2011 and is updated annually to account for inflation [13]. Countries enter a transition process and start phasing out of Gavi support once their GNI exceeds the threshold. The policies for eligibility in the 2021–2025 period (Gavi 5.0) are being modified to account for the reality that many unimmunized children live in middle-income countries (MICs) and that an increasing number of children live in crisis-affected, fragile, and vulnerable settings.

### The Global Vaccine Action Plan

The Global Vaccine Action Plan (GVAP) was a global strategy developed in response to a call in 2010 for a Decade of Vaccines [14]. GVAP was built upon the WHO and UNICEF Global Immunization and Vaccine Strategy (GIVS, 2006–2015), the first 10-year strategy for immunization. GVAP (2011–2020) expanded the partnership, provided a framework for achieving “a world in which all individuals and communities enjoy lives free from vaccine-preventable diseases,” and established measurable goals and targets for the decade [15]. The aspirational goals were to stop wild polio transmission; eliminate neonatal tetanus, measles, rubella, and congenital rubella syndrome; reach 90% national DTP3 coverage and 80% in every district; develop and introduce new vaccines and technologies; and reduce child mortality. One of the first global health strategies to be accompanied by a monitoring and accountability framework with an independent assessment of progress, GVAP achieved substantial progress.

## PROGRESS WITH ACHIEVING GLOBAL GOALS

GVAP demonstrated that coordination and alignment of immunization partners are critical for attaining the aspirations of the decade. Even with these elements, attaining all goals remained elusive and only the target for introduction of new vaccines was met (goal 4) [16]. Nevertheless, enormous advances were achieved and form the basis for the next decade’s strategy.

### Progress Towards Disease Eradication and Elimination

Through the global polio eradication efforts, cases of paralytic poliomyelitis decreased from 350 000 in 1988 to 140 in 2020, after touching a historic low of 22 cases in 2017 [17]. Of the 3 strains of wild poliovirus, type 2 was officially certified as globally eradicated in 2015 and type 3 in 2019 [17]. With the certification of the African continent as being free of all wild-type polio circulation in August 2020, only 2 countries, Pakistan and Afghanistan, continue to have endemic wild-type poliomyelitis transmission. Despite these impressive achievements, the final goal of eradication has remained stubbornly elusive. Recent hurdles have included an upswing in cases of wild-type 1 polio in Pakistan and Afghanistan (84 and 56 cases, respectively, in 2020) due to insecurity hampering routine and supplemental polio vaccination efforts.

Energized by the certification of the eradication of type 2 wild poliovirus in 2015, the Global Polio Eradication Initiative called for the withdrawal of oral polio vaccines starting with the type 2 component of oral polio vaccine (OPV2). This was achieved through a globally synchronized initiative referred to as the “Switch,” in which 156 countries and territories still using oral polio vaccine simultaneously switched from the use of trivalent OPV (tOPV; containing types 1, 2, and 3 poliovirus) to bivalent OPV (bOPV; containing types 1 and 3 poliovirus), in a 2-week time-period, thus removing OPV type 2 from routine use. The global Switch was an unprecedented endeavor and successful milestone for the program, and was accompanied by the introduction of at least 1 dose of inactivated polio vaccine (IPV) in the 126 countries that had an OPV-only schedule. However, since 2016, several challenges have confronted the polio program, including global IPV shortages that contributed to lower than desired coverage in countries where the vaccine was introduced, and months of delay in IPV introduction in many countries. Additionally, the emergence of circulating type 2 vaccine-derived poliovirus (cVDPV2) cases occurred quickly after the Switch, requiring expansive and repeated deployment of monovalent OPV2 from the global stockpile for outbreak response, which has reseeded type 2 poliovirus (vaccine derived) in communities with type 2 immunity gaps, creating a vicious cycle spreading and escalating cVDPVs over the past 2 years. In 2020, there were over 1087 AFP cases due to cVDPVs reported from 24 countries [18]. It is hoped that the implementation of the new cVDPV2 control strategy in early 2021—a key component of which is the roll-out of an improved version of monovalent OPV2 (novel OPV2) that is less likely to seed new outbreaks—will reverse this trend [19]. Despite its limitations, the lessons and experience from IPV introduction, which was the largest and fastest globally coordinated effort for vaccine introduction, has been leveraged for COVID-19 vaccine planning and introduction.

Prior to the introduction of measles vaccine in 1963, measles caused an estimated 2.6 million deaths annually [20]. This fell to 535 600 deaths by 2000; since then and through 2019, an estimated 25.5 million measles deaths have been averted by measles vaccination [21]. Estimated first-dose coverage of measles-containing vaccine increased globally from 72% to 84% over 2000 to 2010; however, between 2011 and 2019 it has plateaued at 84%–85%. Over 19 million children did not receive measles vaccination in 2019 [21]. The global number of reported measles cases (which represent a mere fraction of actual annual measles cases because of underreporting) more than quadrupled from 170 000 in 2017 to 863 000 in 2019, with several countries experiencing large outbreaks, most notably the Democratic Republic of Congo and Madagascar [22]. The Americas, the only WHO Region to verify the elimination of measles (in 2016), lost its elimination status in 2018 from reestablishment of endemic transmission that persisted for more than a 12-month period in some Latin American countries; the other 5 WHO regions have not achieved their time-bound elimination goals, which are now being reset.

The maternal and neonatal tetanus initiative, launched in 1999, targeted 59 endemic countries aiming to reduce neonatal tetanus incidence to < 1/1000 live births in every district of each country [23]. By 2019, 47 (80%) of the 59 target countries had achieved elimination and the estimated number of neonatal tetanus deaths decreased by 85%, from about 171 000 in 2000 to 25 000 in 2018 [23]. Neonatal tetanus afflicts the most marginalized populations, signaling that harsh societal and economic inequities still need to be overcome.

### Immunization Coverage

**Figure 3.**
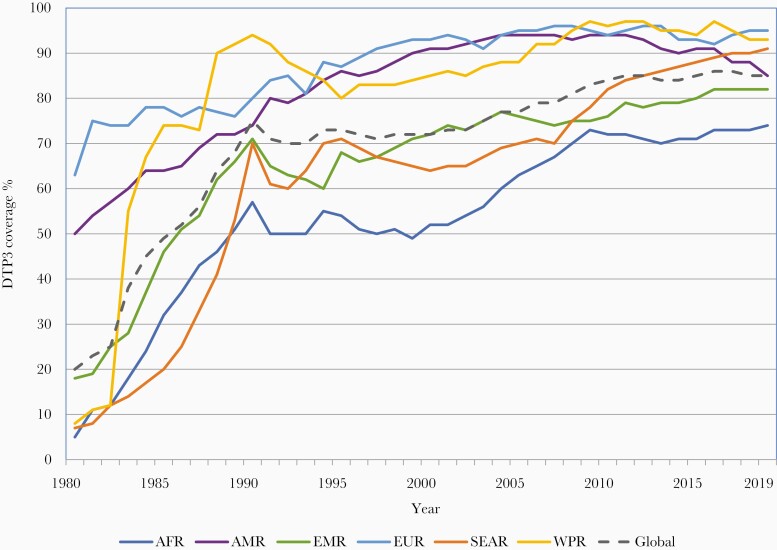
Three doses of diphtheria-tetanus-pertussis vaccine (DTP3) coverage by World Health Organization (WHO) region, 1980 to 2019. Abbreviations of WHO regions: AFR, Africa; AMR, the Americas; EMR, Eastern Mediterranean; EUR, Europe; SEAR, South-East Asia; WPR, Western Pacific.

**Figure 4.**
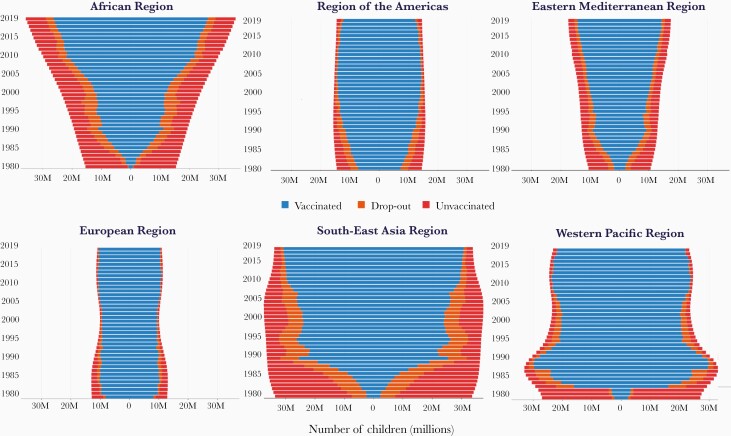
Number of children (in millions) younger than 2 years with 3 doses of diphtheria-tetanus-pertussis vaccine (DTP3), dropout before DTP3, and unvaccinated with DTP1, by region 1980–2019. Abbreviations of World Health Organization regions: AFR, Africa; AMR, the Americas; EMR, Eastern Mediterranean; EUR, Europe; SEAR, South-East Asia; WPR, Western Pacific.

DTP3 coverage is used as a marker of overall immunization program performance because DTP is universally recommended and coverage with 3 doses of DTP permits standardized monitoring over time and across countries. The massive equity gap between the rich and poor countries in 1980 has been markedly reduced; however, since 2000, DTP3 global coverage has remained stagnant between 84% and 86% (Figure 1). In 2019, approximately 85% of infants worldwide (116 million infants) received 3 doses of DTP-containing vaccines [24]. However, the static global coverage masks a highly dynamic situation with impressive gains in some countries and troubling backsliding of coverage in others related to various issues including safety events and political, social, and economic turmoil [25]. There is no guarantee that immunization coverage gains of 1 year are sustained into the next. From a regional perspective, since 2015, DTP3 has steadily increased in the WHO African and South-East Asia regions while there has been a notable decline in coverage in the region of the Americas (Figure 3). The increase in coverage in the African region is especially notable because of the growing birth cohort; to just maintain coverage has required substantial program expansion and increased performance (Figure 4).

### Introduction of New Vaccines

**Figure 5.**
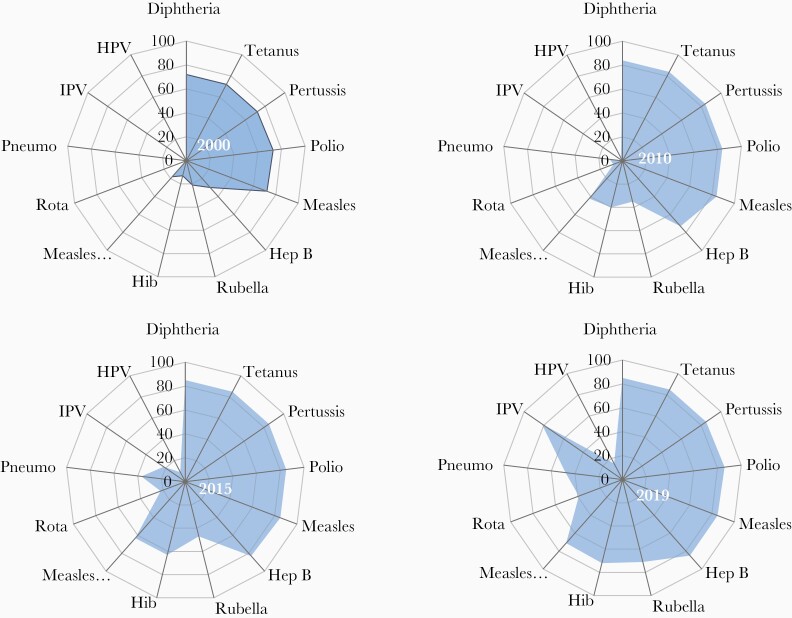
Changes in the breadth of vaccination coverage, 2000 to 2019. The global average coverage for 13 antigens provided through national immunization programs (World Health Organization United Nations Children’s Fund Estimates of National Immunization Coverage). Abbreviations: Hep B, hepatitis B; Hib, *Haemophilus influenzae* type b; HPV, human papilloma virus; IPV, inactivated polio vaccine; Pneumo, pneumococcus; Rota, rotavirus.

Since 2000, the number of vaccines included in childhood immunization schedules has increased dramatically. The historic time lag in the introduction of new vaccines between rich and poor countries has been shortened. Consequently, while DTP3 coverage has remained flat since 2000, the breadth of protection, reflecting the number of antigens and their coverage, has increased substantially (Figure 5). Furthermore, immunization programs now include vaccinations beyond infancy to protect older age groups. These include provision of the second dose of measles-containing vaccines in the second year of life (MCV2) or later, booster doses of DTP vaccine in preschool and school-age children, the introduction of HPV vaccines in the preadolescent and adolescent age group, and seasonal influenza, pneumococcal, and herpes zoster vaccines in older adults, and now COVID-19 vaccines. As of 2019, 178 countries were providing MCV2 in the second to fifth year of life, 106 were providing HPV to preadolescent and adolescent girls, and 112, 25, and 10 were providing seasonal influenza, pneumococcal, and herpes zoster vaccination, respectively, to older adults [26]. Pregnant women are an increasing focus for vaccination, as a means to protect their vulnerabilities and to transplacentally transfer immunity to protect their newborn infants. This advances not only vaccination programs for pregnant women against tetanus, influenza, and pertussis for which recommendations exist, but also advances the interests of pregnant women in vaccine research, especially for emerging diseases [27–28]. Nevertheless, the roll-out of new vaccines has been uneven. Introduction of rubella vaccines was delayed due to concerns about a paradoxical increase in congenital rubella in countries with suboptimal coverage, provision of a timely birth dose of hepatitis B vaccine has been impeded in communities where a sizeable proportion of births occur outside the health facility, and the introduction of HPV vaccines is likely to be affected by vaccine supply shortfalls.

## FACTORS CONTRIBUTING TO SUCCESS

Many factors contributed to the achievements in immunization over the past decades. Improvements in delivery infrastructure; vaccine-preventable disease surveillance and the requisite regional and global lab networks; ever growing community engagement and advocacy on the value of vaccines; more sophisticated information and social media platforms; electronic data collection, management, and use; vaccine research and development capacity; expansion and coordination of vaccine regulatory and safety monitoring systems; increasingly data-driven policy decision making at the country level; and global partnership all underpin this progress. Here we highlight a few factors that are less scientifically driven, yet essential to fully realize the promise of vaccines.

### Commitments by National Governments

National government commitment to prioritizing immunization programs is widely recognized as one of the most impactful success factors without which other efforts falter. Many LMICs have substantially enhanced their commitments to immunization. In 2011, national health ministers in the WHO South-East Asia region expressed their commitment through the Delhi Call for Action to intensify the drive toward achieving high routine immunization coverage throughout the region [29]. Similarly, in 2017, the heads of states of countries in the African continent endorsed the Addis Declaration at the 28th African Union Summit, pledging to ensure that everyone in Africa—regardless of who they are or where they live—receives the full benefits of immunization [30]. The commitments of national governments are reflected by increases in national expenditures on immunization. These increased by 35% in 2016–2017 compared to 2010–2011; they were greatest in the African (74%), South-East Asian (62%), and Western Pacific regions (75%) [31]. Countries also demonstrated their commitment by establishing and/or substantially strengthening national immunization technical advisory groups (NITAGs) to ensure that policymaking on the use of vaccines was driven by evidence. NITAGs meeting all the WHO functionality criteria nearly tripled, from 41 in 2010 to 114 in 2018 [16]. Furthermore, all regions now have Regional Immunization Technical Advisory Groups to adapt global policies to regional contexts, and support NITAGs in adapting and applying these policies at the national level.

### Affordable Pricing and Sustained Supply of Vaccines

Ensuring an adequate supply of safe and effective vaccines remains a global priority in a world with unprecedented changes in the vaccine industry and market [32–34]. LMIC manufacturers have an increasing role in vaccine supply, and their entrance into the market is stimulating price declines of key vaccines [35]. More than 60% of the original 6 EPI vaccines are supplied by LMIC manufacturers [36], who are increasingly entering into development or technology transfer agreements with multinational manufacturers [37].

Predictable financing and innovative procurement mechanisms managed through UNICEF, Gavi, and the Pan American Health Organization Revolving Fund have further contributed to successful tiered pricing mechanisms and substantially reduced pricing for newer vaccines. The weighted average price of the pentavalent vaccine (containing diphtheria, tetanus, pertussis, hepatitis B, and Hib vaccines) for Gavi countries declined from US$2.98 in 2010 to US$0.79 in 2019 [16]. The Vaccine Product, Price and Procurement (V3P) project, and a web-based platform on Market Information for Access (MI4A) have improved price transparency and sustainable access to vaccines at affordable prices [38].

### Improvements in Supply Chains and Logistics

Supply chain and logistics systems that strive to ensure the uninterrupted availability of high-quality and potent vaccines up to the point of administration are the delivery backbone to a well-functioning immunization system, as referred to in WHO’s Immunization Practice Advisory Committee 2014 call to action [39]. The regular monitoring of vaccine stock levels to mitigate stock-outs and address root causes has reduced the risk of interruptions in vaccination services and consequent decline in vaccination coverage in many countries [40]. While few LMICs currently meet the minimum performance standard for every dimension at all levels of the immunization supply chain, as measured through the WHO and UNICEF Effective Vaccine Management assessment process [41], investments by national governments for strengthening their supply chain and establishing robust logistic management information systems are gradually growing.

### Strategies to Reach the Unimmunized

While strong service delivery systems are essential for achieving high and equitable vaccination coverage, these systems need to be accompanied by appropriate policies and strategies that promote the equitable and timely delivery of vaccination. The WHO Global Routine Immunization Strategies and Practices (GRISP) lays out in a comprehensive manner 9 transformative investments that enable resilient programs [42]. GRISP promotes approaches that go beyond conventional service delivery through health facilities and outreach services for detecting and reaching marginalized and partially served communities. These include the Periodic Intensification of Routine Immunization, a mechanism to catch-up individuals who may have missed their routine doses [43]. The WHO Reaching Every District strategy aims to support countries in achieving at least 80% immunization coverage in every district and at least 90% nationally through improved microplanning at the district level [44]. Reducing missed opportunities for vaccination by using all contacts between an individual and the health system is another approach that is being adopted by countries to improve vaccination coverage across the life course [45].

## ADDRESSING THE CHALLENGES FOR GLOBAL IMMUNIZATION

While there has been measurable progress in the current decade, several challenges have impeded progress. Additional efforts, leveraging available opportunities, are needed to realize the new global vision and impact goals of the IA2030 and achieve the 2030 Sustainable Development Goals [46].

### Stagnant and Inequitable Coverage

Achieving and sustaining high and equitable vaccination coverage is fundamental to securing the greatest impact possible with existing and new vaccines. Despite progress in several countries that have had longstanding low vaccination coverage, many have difficulties in equitably reaching and sustaining the 90% target. Several issues merit consideration. In 2019, 20 million children were un- or underimmunized with DTP3, and 14 million of these did not even receive the first dose of DTP, referred to as “zero dose” children [25]. These children are not randomly distributed around the world; over 60% of them are in just 10 countries, some of which have low vaccination coverage (eg, Nigeria and Angola) while others have high coverage but large birth cohorts contributing a large number of undervaccinated children (eg, India and Indonesia). Within countries, these children are from families and communities most likely also to be left out from other essential health services. They are disproportionately those who are impoverished, in rural areas and urban slums, or live in settings of conflict, fragility, or vulnerability [47].

### Policies Lacking an Equity Focus

Existing legislation, policies, and guidelines may inadvertently create or exacerbate inequities. Policies that limit services to individuals registered with civil authorities in a country or region can create barriers for refugee, mobile, or migrant populations, excluding them from access to public health services, including immunization. Gender strongly influences achieving immunization program goals in many settings. A myriad of social and policy structures, such as the ability of women to seek immunization services without having to be accompanied, the gender of immunization workers going door to door, or services that also meet the health needs of women coming for their child’s immunization services are all examples. Examining existing laws and policies through an equity lens will reveal opportunities to address inequities through enactment of new legislation, revision of legal frameworks, and establishment of new policies [48].

### Human Resource Gaps

The positive correlation between health care worker density and immunization coverage is well documented [49–51]. Nevertheless, public health stakeholders and many governments have been unsuccessful in resolving their health worker shortages. Solutions are not straight forward; considerable time, effort, and money are required to train and retain health workers. As immunization programs increase in complexity, the skills and competencies of health workers must also develop. WHO’s Standard Competencies Framework for the Immunization Workforce provides guidance for countries to strengthen human resource capacity to manage and implement their programs [52].

### Inadequate and Unpredictable Financing

The addition of new, more expensive vaccines to EPI has led to increases in the cost of a full vaccination course for a child since 1974; in the last decade alone, the average global immunization expenditures per live birth has increased by 50% [31]. Access to sufficient and predictable financing is essential for immunization programs to sustain vaccination coverage, service quality, and access to newer vaccines [15]. Although national governments in LMICs are increasing the allocations of domestic resources for their immunization programs, dependency on external support is projected to continue in the coming years [53]. In countries benefiting from Gavi support, absolute government investments have increased, but government financing accounted for only 37% of total immunization expenditures in 2017, which represents a 46% decline compared to 2010 because expenditures have outpaced absolute increases in domestic financing [40].

While government health budgets will remain the primary sources of immunization financing, new financing mechanisms need to be explored if LMICs are to sustain their immunization programs. As part of their efforts towards achieving universal health coverage, several new mechanisms are being used or considered for the costs of delivering an essential package of health services. An immunization financing resource guide provides options for raising additional resources to support immunization programs and the use of social insurance to partly finance immunization programs [54].

### Uneven Access to Vaccines at Affordable Prices

**Figure 6.**
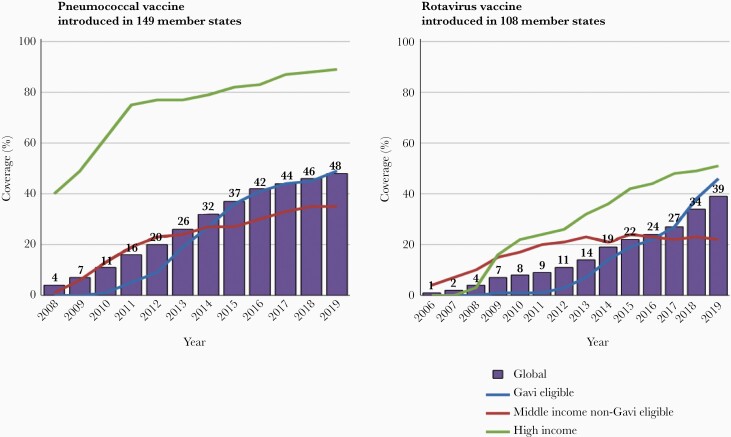
Trends in coverage with pneumococcal conjugate and rotavirus vaccines by country income levels and eligibility for Gavi support. Coverage in each country group is the product of the number of countries in the group that have introduced the vaccine and the coverage achieved nationally.

The uptake of new vaccines in MICs that are not eligible for Gavi support has been slower than in Gavi-eligible countries [55]. In fact, coverage of pneumococcal and rotavirus vaccines is now higher in Gavi countries than the global average because many MICs have yet to introduce these vaccines (Figure 6). Available information suggests that there is a wide range of prices paid by MICs for the same vaccine products. Previous studies have identified inefficient procurement as a barrier to competitive prices for vaccines [56]. While the market intelligence and price transparency provided by the WHO’s V3P and MI4A platforms have made an important contribution, improvements to country regulatory procedures and procurement practices, which contribute to higher pricing, has been patchy and more is required to secure affordable price access.

### Limitations in the Quality and Use of Immunization and Surveillance Data

A major obstacle to closing current immunization gaps has been the lack of timely and high-quality data to inform decision making, operational planning, performance management of immunization staff, and service delivery. This information is essential at all levels of the health system for the effective delivery of immunization services. The absence of such data has consequences including missed opportunities to identify under- or unimmunized persons, inadequate defaulter tracking, and burdensome data collection processes that divert health provider time and attention away from the provision of high-quality service delivery. WHO’s Strategic Advisory Group of Experts on Immunization (SAGE) highlighted these issues and recommended specific actions to improve the quality and use of data [57].

#### Coverage Monitoring

Inaccuracies in vaccination coverage estimates result from imprecise estimates of the target population size (the denominator) and errors in the recording of immunization doses administered (the numerator). In many LMICs, estimates of the target population size are from national census data, which are often very outdated. Despite the well-documented benefits of civil registration and vital statistic systems (CRVS), countries have been slow to adopt these systems. WHO guidelines and tools are available to conduct comprehensive assessment of CRVS and to strengthen them [58]. Several LMICs have established national plans based on these comprehensive assessments.

An increasing number of LMICs are leveraging the availability of new information and communication technologies to update their immunization data management systems that would improve the recording, timely transmission, and use of data to monitor their programs. Electronic immunization registries (EIRs) are computerized, confidential, population-based systems that contain individual-level information on vaccine doses administered [59, 60]. An increasing number of high and middle-income countries have, or are establishing, EIRs [59–61], while a few LMICs have received support to establish or pilot EIRs [62].

Many other LMICs are using the District Health Information Software 2 (DHIS2), an open source, web-based health management information system platform, to collect and manage health and immunization data [63]. DHIS2 has developed an e-tracker that enables collection, management, and analysis of transactional, case-based data records, supports storage of information about individuals, and tracks these persons over time using a flexible set of identifiers. This system was rolled out in Rwanda in 2019 as an EIR [62]. It has recently been adapted to support the COVID-19 vaccine introduction monitoring, including a safety reporting module.

#### Disease and safety surveillance

Surveillance is required to measure disease burden, quantify the impact of immunization programs in reducing vaccine-preventable diseases, and identify changes in disease epidemiology that may require modifications of vaccination schedules and/or adaptation of program strategies. Disease surveillance data are also key for informing policy decisions and supporting sustained financing for the immunization program. The surveillance systems in many LMICs remain fragmented into disease-specific surveillance initiatives largely dependent on external resources. A shift to country-owned, comprehensive disease surveillance is needed to address the fragmentation, improve efficiencies, and ultimately lead to sustainable surveillance as is laid out in WHO’s new global strategy for comprehensive vaccine-preventable disease surveillance [64].

Funding for disease surveillance activities has leaned heavily on the Global Polio Eradication Initiative for many years and is expected to wind down over this decade. Alternate funding, including more country-level commitment to surveillance, will be needed. An investment case for surveillance in the African region indicates that if current vaccine-preventable disease surveillance and laboratory networks are enhanced, there would be an estimated 45-fold return on investment by 2030 [65].

Safety monitoring and surveillance for adverse events following immunization are key components of a strong immunization program. The WHO Global Vaccine Safety Blueprint aims to optimize the safety of vaccines through effective use of pharmacovigilance principles and methods and assist low and LMICs to have at least minimal capacity for vaccine safety activities [66].

### Wavering Community Demand for Vaccination

It is naive to assume that if health services are made accessible to communities, that health-seeking behavior will follow [67]. Analysis of data reported to WHO and UNICEF for 2015 to 2017 showed that vaccine hesitancy, defined as “delay in acceptance or refusal of vaccination despite availability of vaccination services,” was common; over 90% of countries reported encountering hesitancy in accepting vaccination [68]. This led WHO to include vaccine hesitancy among 10 threats to global health in 2019 [69]. There were varied reasons cited for hesitancy with heterogeneity across countries [68]. A recent review found that while confidence in vaccines fell in some countries, it improved in others, highlighting the need for continuous monitoring and corrective actions [70]. However, demand for immunization encompasses more than hesitancy alone. It is also misleading to draw a clear distinction between service- or system-side factors and demand-related issues because an individual’s prior experience with the health system may influence their future demand or uptake of services [67]. Given the highly contextual nature of vaccine demand, community engagement and formative research is required to identify the determinants of demand and tailor services to improve the uptake of vaccination at local levels. The Tailoring Immunization Programs [71] and Human Centered Design for Health [67] are tools to enable structured, adaptable and participatory processes to target undervaccinated or hesitant populations using behavioral insights to understand the barriers and enablers of vaccination and to design, implement, and evaluate tailored, gender-responsive interventions to address them [72]. The Vaccination Demand Hub is a network of partner organizations collaborating to understand why people miss out and to improve acceptance and uptake of vaccines through actions to counter the priority barriers [73].

### Emerging Inequities in Middle-Income Countries

MICs account for 60% of global deaths in children younger than 5 years, and a similar or greater share of vaccine-preventable deaths and unvaccinated children [74]. The initial impetus for the focus on MICs in the immunization field was a concern that these countries, excluded from or facing the loss of donor support and insufficient domestic investments in health, may be missing out on opportunities to introduce important new vaccines. However, the problems facing MICs extend far beyond the introduction of newer vaccines. Globally, vaccination coverage in the fully self-financing MICs has declined in recent years while coverage in HICs remained stable and increased in Gavi-supported LMICs (Figure 1). In the European region, the MICs not eligible for Gavi support are lagging behind the HICs and the Gavi-supported MICs in progress with other regional goals [75]. A strategy to support fully self-financing MICs exists but needs the support and resources for its implementation [74].

### Outbreaks, Conflicts, and Humanitarian Emergencies

The COVID-19 pandemic has demonstrated that immunization systems need to have the resilience to rapidly recover from acute shocks, such as during prolonged disease outbreaks and other causes of disruption. Lessons need to be documented from the Global Polio Eradication Initiative and case studies developed from exemplar countries who have overcome the challenges posed by conflict, civil unrest, and other humanitarian emergencies to cultivate best practices on sustaining immunization services during periods of severe disruption. In addition, countries need to improve their preparedness for and capacity to rapidly detect and respond to outbreaks of vaccine-preventable diseases to mitigate the risks of a prolonged epidemic. Conversely, data from outbreaks should be used to identify root causes behind immunity gaps, to address both vaccine supply- and demand-side shortcomings.

### Impact of the COVID-19 Pandemic

**Figure 7.**
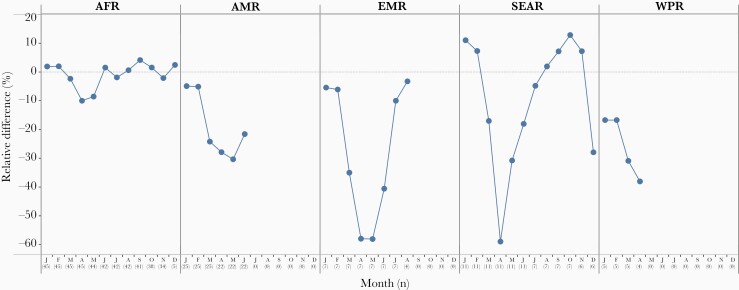
Impact of the COVID-19 pandemic on routine childhood immunization. Relative difference between DTP3 doses administered in 2020–2021 and the similar time period in 2019 in 5 of the 6 WHO regions. Abbreviations: AFR, Africa; AMR, Americas; COVID-19, coronavirus disease 2019; DTP3, 3 doses of diphtheria-tetanus-pertussis vaccine; EMR, Eastern Mediterranean; SEAR, South-East Asia; WHO, World Health Organization; WPR, Western Pacific. Source: monthly administrative data on third dose of DTP administered reported to WHO as of 4 March 2021.

The COVID-19 pandemic has revealed the vulnerability of the immunization program, an essential health service. In the first quarter of 2020, sharp declines in immunization coverage, ranging from 10% to 50% relative to 2019, occurred as severe social and physical distancing policies were implemented in many countries around the world (Figure 7). During this same period, preventive polio, measles, and other vaccine campaigns were temporarily suspended due to the concern for COVID-19 transmission in campaign settings. Cumulatively as of 15 October 2020, 91 vaccination campaigns in 53 countries had been postponed including for polio, measles, rubella, tetanus, diphtheria, cholera, typhoid, meningitis, and yellow fever. Within 3 months of the onset of vaccine program disruptions, countries sought and implemented adaptations and innovations with the aim to restore and recover essential immunization services, including for outbreak response and preventive campaigns. New ways of working, such as offering immunization services in novel locations, drive-through vaccination, expanding service days and hours, providing appointment times rather than open clinic hours, and leveraging social media have all mitigated the impact of the pandemic. As a result, several regions showed a V-shaped recovery in vaccination service delivery (Figure 7). By December 2020, 57 vaccination campaigns that had been postponed were implemented. Nevertheless, as countries phase out of lock-down measures, implementation of aggressive catch-up strategies will be required to immunize those who have missed their scheduled immunizations, to prevent risks of vaccine-preventable disease outbreaks such as measles [76].

## LOOKING TO THE FUTURE

### A COVID-19 Vaccines Roll Out

An unprecedented worldwide effort began in January 2020 to develop safe and effective COVID-19 vaccines to help end the COVID-19 pandemic that has impacted the lives and livelihoods of people worldwide. Following interim phase 3 clinical trial results and authorization for emergency use, COVID-19 vaccines began rollout in December 2020. This is an unprecedented landmark in immunization and shows how this pandemic has created a paradigm shift in the process, speed and scale vaccines are being developed, deployed, and financed. The goal of facilitating fair and equitable access to COVID-19 vaccines has motivated the establishment of the COVAX Global Vaccine Facility as the vaccine pillar of the Access to COVID Tools Accelerator. The Facility, co-led by WHO, Gavi, and the Coalition for Epidemic Preparedness Innovations in partnership with UNICEF and others, aims to assure speed, scale, and equitable access to COVID-19 vaccines with demonstrated efficacy and safety [77].

The WHO target product profile for COVID-19 vaccines provides a benchmark of the minimum characteristics needed for the assessment of candidates [78]. There are around 264 vaccine candidates in development as of 21 March 2021 of which 20 are in or entering phase 3 clinical trials [79]. An allocation framework for equitable distribution of vaccines across countries has been developed by WHO in consultation with its member states [80]. A values framework that articulates the ethical principles through which vaccine allocation and prioritization should be made, and a roadmap for prioritization of limited vaccine supply within countries to target populations, has been issued by SAGE and endorsed by WHO [81, 82]. SAGE is providing product specific recommendations for any COVID-19 vaccine with emergency use listing by WHO or authorization by a WHO-recognized stringent regulatory authority.

### Leveraging the Power of Research and Innovation

New technologies and innovative strategies have the potential to increase the reach and impact of immunization programs. While research has led to the development of new life-saving vaccines, research to develop new or improved products better suited to local needs, implementation research on how to optimize immunization among underserved populations, and to develop, evaluate, and scale up innovations for immunization service delivery could amplify the impact of available products as well as those in the pipeline. One example of targeting innovations to local needs is The Vaccine Innovation Prioritization Strategy, which undertook a formal process, including engaging in-country stakeholders, to prioritize 3 vaccine product innovations (among 24) with the greatest potential to achieve equity, improve immunization systems, and focus investment; these 3 innovations are microarray patches, heat-stable controlled temperature chain liquid formulations, and barcoding on primary containers [83]. However, research needs to extend beyond the development and launch of innovative products to address managerial, systems, sociobehavioral, financial, and communications bottlenecks. Implementation and operational research is one of the key focus areas in the IA2030 [5].

### Catalyzing the Move Towards Life-Course Vaccination

Immunization beyond the infant schedule has not yet reached the scale of implementation it merits. The reasons to build strong immunization services throughout the life course are many [84]. First, to deliver booster and missed doses to achieve durable individual protection and herd immunity, and thereby achieve better disease control. Second, for protection against diseases with higher risk of transmission or more severe morbidity during pregnancy, in health care workers, travelers, in older ages, or for medical risk groups. To establish and maintain life-course immunization, policy and legal frameworks need to be adapted, systems to monitor immunization in older age groups expanded, and collaborations to integrate age-appropriate and catch-up vaccination into public and private health services strengthened. COVID-19 vaccine delivery will be an opportunity to strengthen adult vaccination approaches and integration with other health care programs, because the target groups include health care workers, adults of older age, and medical risk groups.

### The Immunization Agenda 2030

In August 2020, all WHO member states endorsed the Immunization Agenda 2030: A Global Strategy to Leave No One Behind (IA2030) [5]. IA2030 envisions “a world where everyone, everywhere, at every age, fully benefits from vaccines to improve health and well-being.” It was developed in a co-creative process with close engagement of countries to ensure that the vision, strategic priorities, and goals are aligned with country needs. During its development, collective input came from over 60 organizations and in-person global and regional workshops of more than 750 individuals. The agenda is organized into 7 strategic priorities for immunization in the next decade: immunization programs for primary health care and universal health coverage; demand and commitment; coverage and equity; life course and integration; outbreaks and emergencies; supply and financing; and research and innovation. Four core principles—people-centered, country-owned, partnership, and data-guided—underpin all strategic priorities and are essential to its success.

IA2030 focuses on tailored implementation and adaptive approaches to country contexts and is built to be flexible to new challenges throughout the decade. The main shift from GVAP is a move away from disease-specific ways of operating and place immunization as an integral part of strong people-centered primary health care service. The agenda places countries at the center, supported by accountable partners including those outside the health sector. Other shifts include the importance of data-driven program and policy improvements, and the importance placed on targeted ways of addressing inequities and gender-related barriers. Measles being the most contagious vaccine-preventable disease, is recognized as a pathfinder for IA2030, signaling when and where immunization services need to be improved. To operationalize IA2030, regional IA2030 plans are being developed and countries are integrating the strategies into national immunization strategies aligned with the public health plans in each country. The monitoring and accountability framework is being developed through a wide country consultation process and will be presented to the World Health Assembly in May 2021. The IA2030 offers a valuable platform for sustaining global commitment and promoting greater accountability for improved health and well-being.

## Notes

***Acknowledgments.*** The authors gratefully acknowledge the contributions of the following staff from the Department of Immunization, Vaccines, and Biologicals and Global Polio Eradication Initiative, WHO, Geneva, and from the Immunization Departments at the WHO Regional Offices in providing information and data for preparing the manuscript: Sébastian Antoni, Andre Arsene Bita Fouda, Sunil Bahl, Nyambat Batmunkh, Kelly Carr, Natasha Crowcroft, Carolina Danovaro, Siddhartha Datta, Laure Dumolard, Kamal Fahmy, Marta Gacic-Dobo, Randie Gibson, Jan Grevendonk, Santosh Gurung, Quamrul Hasan, Lee Lee Ho, Katrina Kretsinger, Ondrej Mach, Balcha Girma Masresha, Richard Mihigo, Yohann Nedelec, Roberta Pastore, Alain Nyembo Poy, Alejandro Ramirez Gonzalez, Cuauhtemoc Ruiz, Mohammed Sharifuzzaman, Anita Shet, Claudia Steulet, Yoshihiro Takashima, Martha Velandia, and Simona Zipursky.

***Disclaimer.*** The named authors alone are responsible for the views expressed in this publication.

***Supplement sponsorship.*** This supplement is sponsored by the Bill and Melinda Gates Foundation.

***Potential conflicts of interest.*** All authors: No reported conflicts of interest. All authors have submitted the ICMJE Form for Disclosure of Potential Conflicts of Interest. Conflicts that the editors consider relevant to the content of the manuscript have been disclosed.
